# Unveiling zoonotic threats: molecular identification of *Brugia* sp. infection in a lion

**DOI:** 10.3389/fvets.2024.1376208

**Published:** 2024-04-12

**Authors:** Witchuta Junsiri, Patchana Kamkong, Aunchisa Phojun, Piyanan Taweethavonsawat

**Affiliations:** ^1^Parasitology Unit, Department of Veterinary Pathology, Faculty of Veterinary Science, Chulalongkorn University, Bangkok, Thailand; ^2^Nakhon Ratchasima Zoo, The Zoological Park Organization, Nakhon Ratchasima, Thailand; ^3^Biomarkers in Animals Parasitology Research Unit, Chulalongkorn University, Bangkok, Thailand

**Keywords:** *Brugia malayi*, lion, molecular analysis, PCR, Thailand

## Abstract

*Brugia malayi* and *B. pahangi*, potential zoonotic pathogens transmitted by mosquitoes, are believed to primarily infect dogs and cats as reservoir hosts. Although previous studies have indicated nematode infections in lions, particularly in zoo environments where human contact with these reservoirs is possible, limited documentation exists regarding *Brugia* sp. infections in lions in Thailand. This study aims to investigate a case of *Brugia* infection in a lion from a zoo in Thailand. The blood sample was collected and examined from a female lion, using staining methods to morphologically identify microfilaria at the genus level. Subsequently, the PCR was employed targeting specific genes, including mitochondrial 12S rDNA, 18S rDNA, cytochrome oxidase I (COI) and *Wolbachia* surface protein (*wsp*), to confirm the species of the filarial nematode parasite. The genetic sequencing results revealed a high similarity (99–100%) to *B. malayi* for the 12S rDNA, 18S rDNA, COI and *wsp* genes. Phylogenetic analysis based on nucleotide sequences from the 12S rDNA, 18S rDNA, COI and *wsp* genes showed that the sequences from this study belong to different clusters. This marks the inaugural documentation of molecular identification of *Brugia* infection in a lion, signifying that lions could function as reservoirs for this parasite and present a potential public health risk in the region. Our research underscores the effectiveness of molecular techniques and phylogenetic analysis in discerning and comprehending the evolution of filarial parasites. Additionally, it emphasizes the significance of these methods in enhancing the diagnosis, control, and prevention of zoonotic filarial nematode infections.

## 1 Introduction

*Brugia* spp., nematodes of the Onchocercidae family, have a global presence in infecting the lymphatic system of mammals. Lymphatic disease, commonly referred to as elephantiasis, is regarded as the primary manifestation of filariasis (1). Among *Brugia* species of medical importance are *Brugia malayi* and *Brugia timori*, causative agents of lymphatic filariasis in south and southeast Asia (2, 3). *Brugia malayi* can also naturally infect mammals such as monkeys and cats (4–6). Other filarial species, such as *B. pahangi*, have been reported to have associations with domestic animals (7, 8). *Brugia pahangi*, a closely related species of *B. malayi*, is a lymphatic filarial worm of mammals, particularly of cats, dogs and wild carnivores (9, 10). Although there have been reports of the presence of this parasite’s microfilaria in human blood samples, it is not currently identified as a cause of human disease in its natural environment (11, 12).

Filarial nematodes have previously been reported in different species of wild or captive felids, such as *Dirofilaria immitis* has been found in Bengal tiger (*Panthera tigris*) (13, 14), snow leopard (*Uncia uncia*) (15), clouded leopard (*Neofelis nebulosa*) (16), African lion (*Panthera leo*) (17), leopard (*Panthera pardus pardus*) (18). *Dirofilaria striata* has been reported in Florida panthers (*Felis concolor coryi*) and the bobcat (*Lynx rufus*) (19). Genet cats (*Genetta tigrina*) were infected with *D. repens* in East Africa (20). To date, there have been no documented cases of lymphatic filariasis resulting from *Brugia* sp. infection in lions globally, including Thailand. Nevertheless, the result showed an instance of *Brugia* sp. infection in a captive lion from a private zoo in Thailand. Molecular characterization of the infection was achieved through the analysis of the 12S rDNA, 18S rDNA, COI, and *wsp* genes.

## 2 Materials and methods

### 2.1 Sample collection

On October 27, 2022, a routine health examination was conducted on two lions (*Panthera leo*) at a private zoo. Both lions, a 2-year-old male and a 2-year-old female, weighing 90 kg each, were anesthetized for the examination. The lions had a body condition score of 3. Anesthesia was induced using a combination of 1 mg/kg xylazine (X-LAZINE, Thailand) and 4 mg/kg ketamine (Hameln Pharma, United Kingdom). Subsequently, blood samples were drawn from the femoral vein using EDTA tubes. These samples were then sent to a private standard laboratory center (Nakhon Ratchasima Province) to determine the presence of microfilariae through Giemsa staining (21) and Acid phosphatase staining (22). A blood sample was subjected to testing using an FIV/FeLV test kit (IDEXX, United States) and a CPV/CCV test kit (IDEXX, United States). The results revealed that a female lion tested positive for Brugian filariasis. The remaining EDTA blood from the Brugian filariasis-positive lion was preserved at −20°C for subsequent DNA extraction.

### 2.2 DNA extraction and molecular assay

For molecular identification, NucleoSpin^®^ Blood (MACHEREY-NAGEL, Germany) was used to extract filarial nematode DNA according to the manufacturer’s specifications. The DNA was used as a template for the PCRs with the GoTaq^®^ Green Master Mix (Promega Corporation, United States), amplifying a section of the 12S rDNA, 18S rDNA, COI, and *wsp* genes. The cycling conditions for PCR and specific details of primer sequences were employed as outlined in Table 1. All PCR amplifications included *D. immitis* adult worm DNA as an amplification control, and nuclease-free water served as a no-template control. The amplified PCR products were stained with RedSafe™ Nucleic Acid Staining Solution (INtRON Biotechnology, Korea) and checked in 1.5% agarose gel. Further amplified DNA product was excised from the gel and purified using a PCR clean-up gel extraction kit (NucleoSpin^®^ Gel and PCR Clean-up, MACHEREY-NAGEL, Germany) as per the manufacturer’s instructions. Purified PCR products were carried out in both directions and confirmed by barcode taq (BT) sequencing. DNA sequences obtained in the study were identified using BLAST with sequences available in the GenBank database. The 12S rDNA, 18S rDNA, COI and *wsp* sequences obtained from this study were aligned along with the reference sequences retrieved from NCBI using CLUSTALW. Finally, the phylogenetic analysis was performed using MEGA X software (27). The maximum likelihood algorithm was used as the best fit model with 1,000 bootstrap replicates (28) and the nucleotide distance was calculated using the p-distance method (29).

**Table 1:** Primer list for the optimized *Brugia* sp. detection protocol.

Target gene	Primers	Sequence (5′–3′)	Annealing temperature (° C)	Product size (bp)	References
12S rDNA	12sF	GTTCCAGAATAATCGGCTA	52	484	(23)
	12sRdeg	ATTGACGGATGRTTTGTACC			
18S rDNA	18SF	TCGTCATTGCTGCGGTTAAA	54	753	(24)
	18SR	GGTTCAAGCCACTGCGATTAA			
COI	cox1intF	TGATTGGTGGTTT TGGTAA	54	672	(25)
	cox1intR	ATAAGTACGAGTATCAATATC			
*wsp*	WSPF	AACTGCTTTAGTGGCGTTGC	60	723	(26)
	WSPR	TTAAACATTAACCCAGCTTCTGTGC			

## 3 Results

### 3.1 Parasite identification

**Figure 1:**
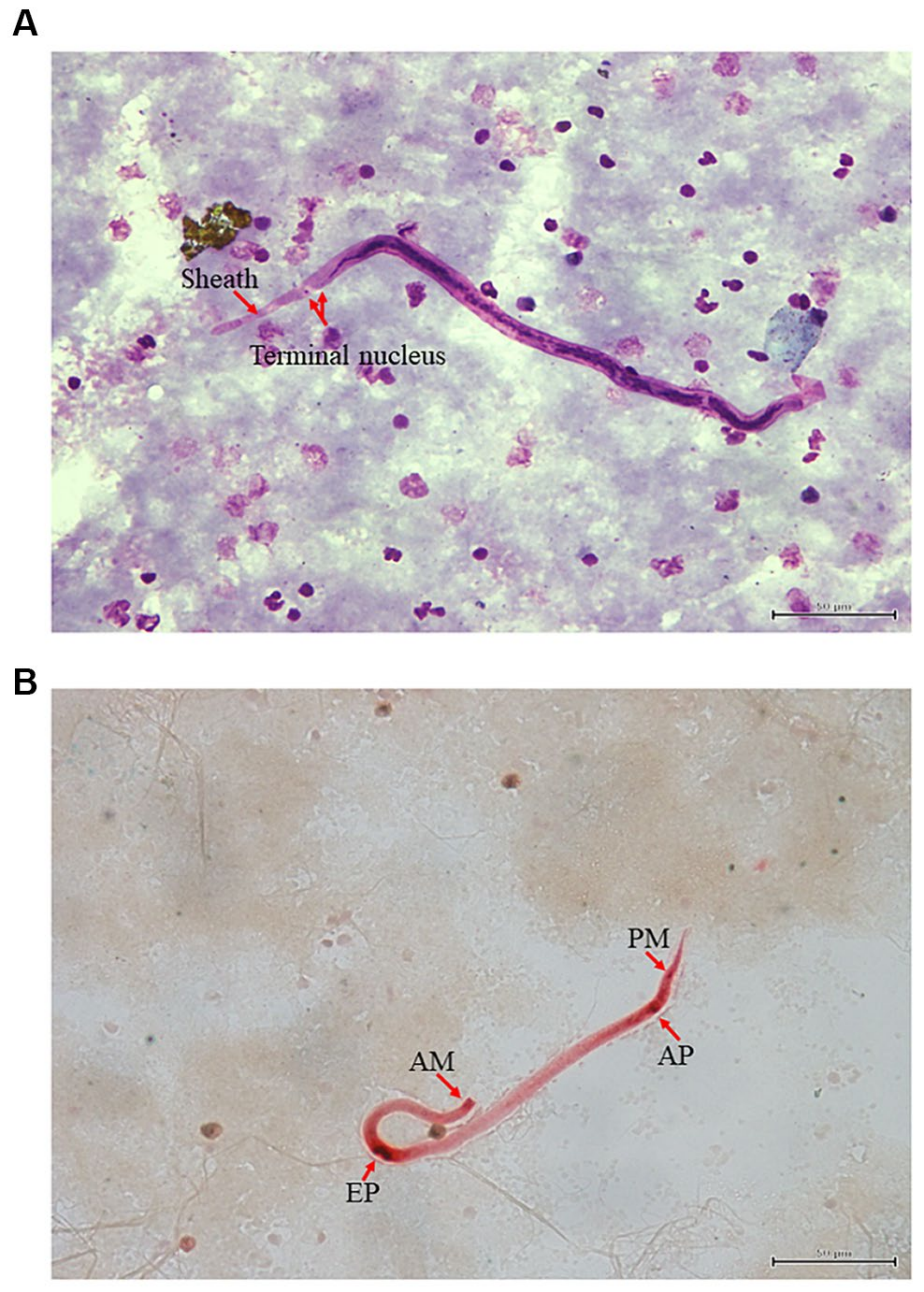
Microscopic examination using Giemsa and Acid phosphatase straining **(A,B)**: Giemsa staining of microfilaria infection from lion (*Panthera leo*) in Thailand. Red arrow indicates the presence of sheathed microfilaria **(A)**. *Brugia* microfilaria showing acid phosphatase activity at the amphid (AM), excretory pore (EP), anal pore (AP) and phasmid (PM) **(B)**.

A 2-year-old female lion blood sample was brought to Vet Central Lab for screening of microfilariae. The Giemsa blood smear taken from the positive case showed the presence of sheathed microfilariae. These sheathed microfilariae had a pink sheath, with a head-space at the front and two clear tail nuclei at the back (Figure 1A). Histochemical staining was performed to confirm species identification by acid phosphatase activity in microfilariae. When the sheathed microfilariae were stained with acid phosphatase, a pattern of four points staining emerged, indicating acid phosphatase activity at the amphid (AM), excretory pore (EP), anal pore (AP), and phasmid (PM) (Figure 1B). When it comes to *B. malayi* microfilaria, bright red points were seen, which were easily visible even under low magnification.

### 3.2 Similarity analysis

The primers used for PCR amplification successfully detected filarial nematode DNA in the blood samples. The *Brugia* sp. was specifically identified through PCR products of various sizes, including 484 bp (12S rDNA gene), 753 bp (18S rDNA gene), 672 bp (COI gene) and 723 bp (*wsp* gene). These PCR products were then sequenced and analyzed using BLAST. The BLAST results demonstrated that the 12S rDNA, 18S rDNA, COI and *wsp* sequences shared a high level of identity (99–100%) with *B. malayi*. The nucleotide sequence data mentioned in the paper have been deposited in GenBank, as indicated in Supplementary Table S1. Comparative analysis of the sequences revealed a high sequence similarity percentage (99.7–100%, 95.2–100%, 99.5–100% and 99.4–100%) for the 12S rDNA, 18S rDNA, COI and *wsp* sequences, respectively (Supplementary Tables S2–S5).

### 3.3 Phylogenetic analysis

**Figure 2:**
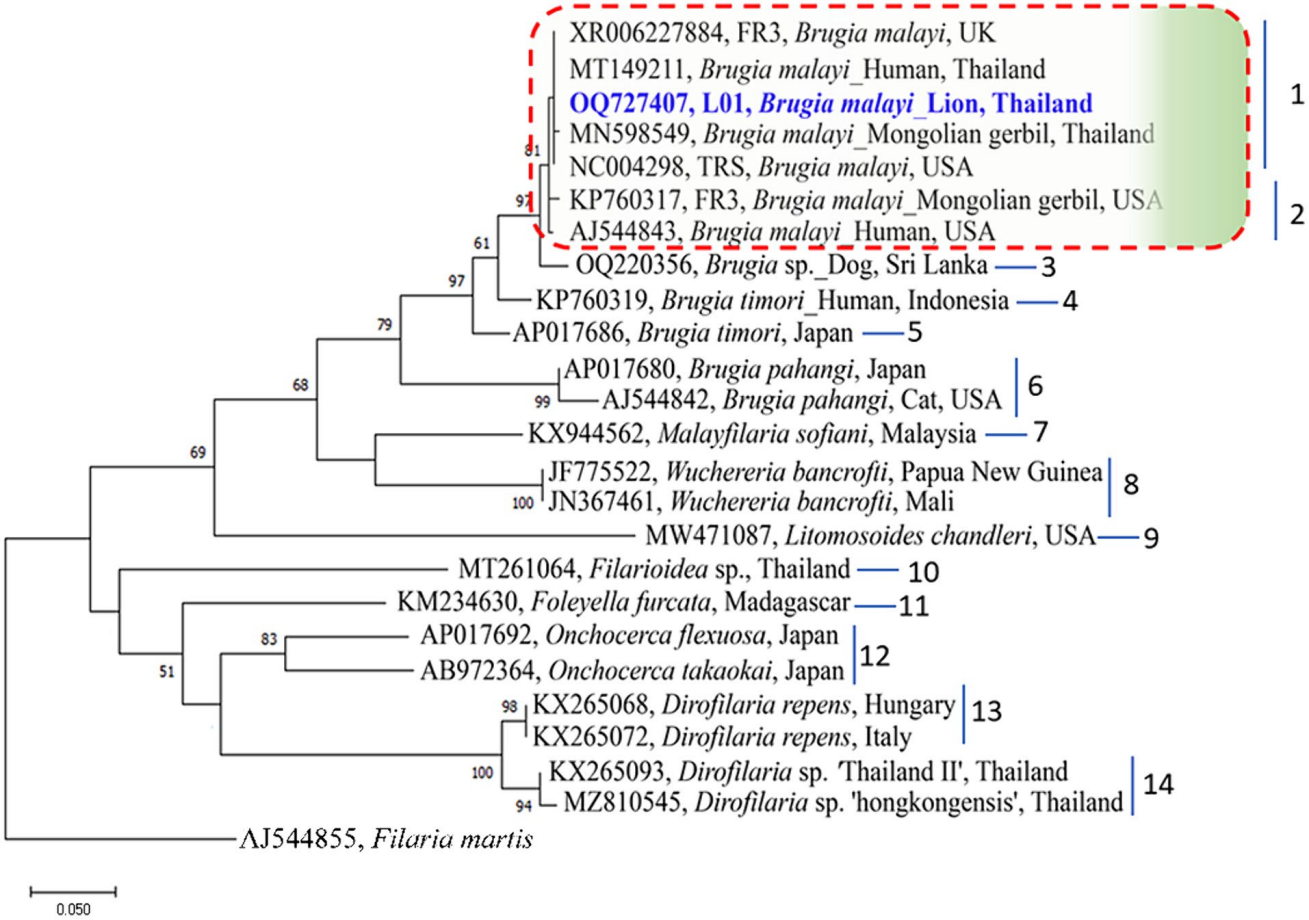
Phylogenetic tree based on an alignment of 12S rDNA gene sequences. Bootstrap confidence values (1,000 replicates) are shown as percentages. Values less than 50% are omitted. The units for the scale bar are substitutions per site. NCBI accession numbers are included. The 12S rDNA sequence of *B. malayi* from lion in Thailand, with taxon name shown in blue color and bold font, was generated as part of this study. Letters to the right of the bracketed branches denote the clusters. *Filaria martis* was used as out group.

**Figure 3:**
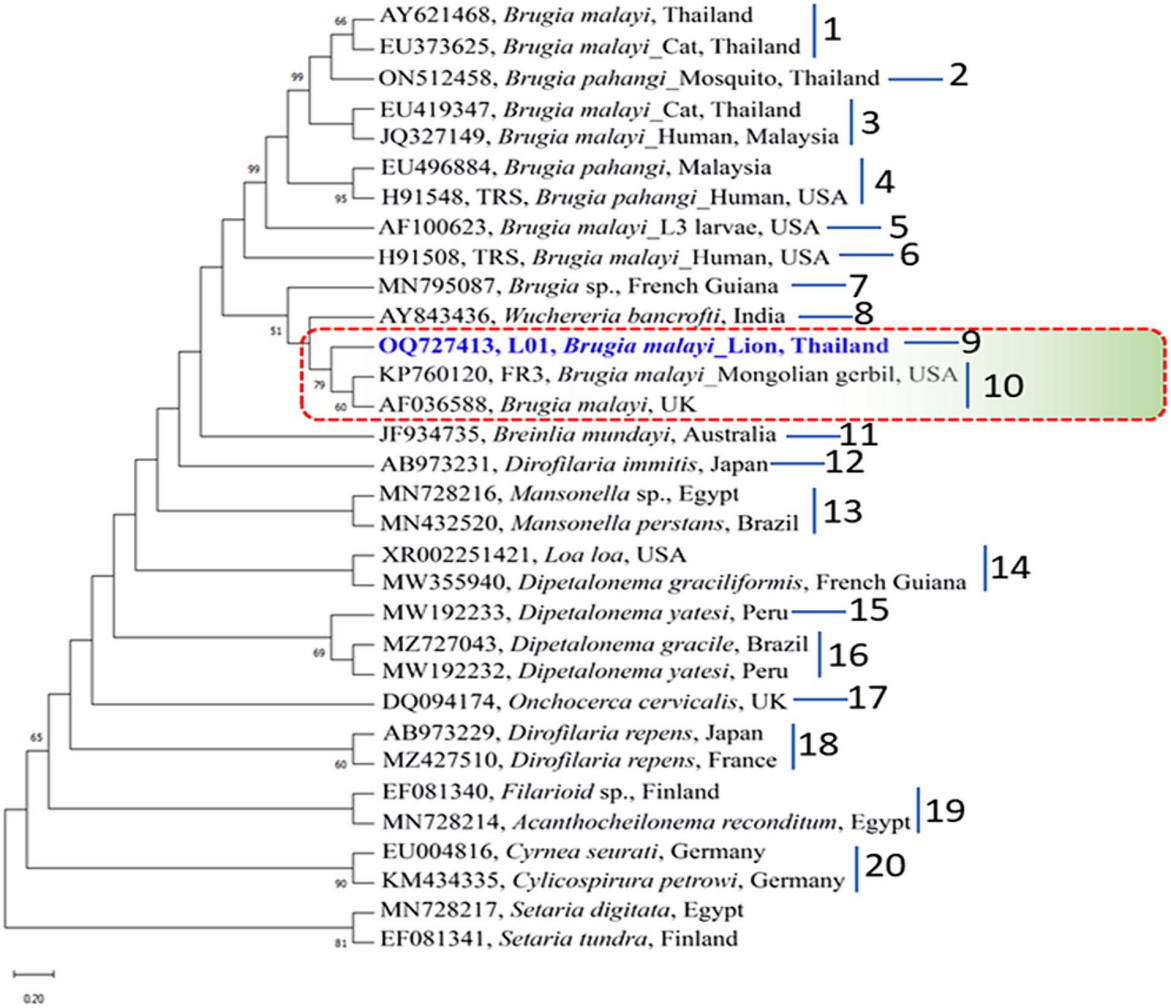
Phylogenetic tree based on an alignment of 18S rDNA gene sequences. Bootstrap confidence values (1,000 replicates) are shown as percentages. Values less than 50% are omitted. The units for the scale bar are substitutions per site. NCBI accession numbers are included. The 18S rDNA sequence of *B. malayi* from lion in Thailand, with taxon name shown in blue color and bold font, was generated as part of this study. Letters to the right of the bracketed branches denote the clusters. *Setaria digitata* and *S. tundra* were used as out group.

The genetic relationships among *B. malayi* isolates can be more accurately determined by representing them in a phylogenetic tree. In our study, the Maximum Likelihood (ML) was used to construct these trees. By aligning the sequences of the *B. malayi* 12S rDNA gene obtained in our study with other sequences from GenBank, divided the resulting phylogenetic tree into 14 clusters. Notably, a *B. malayi* sequence identified in our study was placed in cluster 1 along with sequences from the UK, USA, and Thailand (Figure 2). In the phylogenetic tree of the 18S rDNA gene, the sequences were classified into 20 clusters within the phylogram. Interestingly, a *B. malayi* sequence from a lion in Thailand was assigned to cluster 9 and showed genetic similarities with sequences from the UK and USA, indicating the genetic variability of the *B. malayi* 18S rDNA sequence in Thailand (Figure 3). The COI gene sequences were also analyzed, which were grouped into 8 clusters. A *B. malayi* COI gene sequence from a lion in Thailand was classified into cluster 1, alongside other *B. malayi* COI sequences from Thailand, Vietnam, and the USA (Supplementary Figure S1). Furthermore, the phylogenetic analysis of the *wsp* gene in *Wolbachia* endosymbionts revealed clear separation into 18 clusters. Within the cluster associated with *Brugia* sp., a more detailed subdivision into 9 subclusters was observed (Supplementary Figure S2). The *B. malayi* sequence identified in our investigation was classified within the 1st cluster, exhibiting genetic affinities with sequences originating from the USA.

## 4 Discussion

Presently, there is a lack of literature on *Brugia* infection in zoos and wildlife animals in Thailand. However, to the best of our knowledge, this study is the inaugural one to present both morphological and molecular characterization of *Brugia* sp. infection in captive zoo lions in Thailand. Morphological assessment is employed to identify potential causes, and subsequent molecular characterization aids in pinpointing the most likely parasitic source. Knowledge of lion parasites mainly from zoo lions and mostly focused on intestinal parasites (30, 31). Limited information exists regarding the prevalence of parasites in free-ranging lions (32). Parasites in lions have been documented in various regions, including Tanzania, Ethiopia, South Africa, the USA, Italy, India, and Malaysia (16, 18, 19, 31–34) with a few notable exceptions where a particular group of parasites has been studied, such as blood parasites (35–39), intestinal parasites (32, 40) or where the results of veterinary examinations in a National Park (33) have been reported. A previous study reported that *Dirofilaria* sp. is a common parasite of lions in the Kruger Park (33), while our study found microfilaria of *Brugia* sp. infected in lion. The result of this study similar to result reported by Zahedi et al. (16) who found microfilaria of *Brugia* in a clouded leopard (*Neofelis nebulosa*). However, the study found a mixed infection of *B. pahangi* and *D. immitis*, which differs from our study that reported the infection of *B. malayi*.

The criteria for morphological identification of microfilariae include their size, sheath, and nuclear column (41). Filarial infection with multiple species and morphological alterations of microfilariae are not easily differentiated morphologically even by trained persons (42). Histochemical staining to detect acid phosphatase activity can overcome most of these problems (43). Our result showed the lion microfilariae are sheathed with four points staining pattern with acid phosphatase activity at the amphid (AM), excretory pore (EP), anal pore (AP) and phasmid (PM). Moreover, *B. malayi* microfilaria had bright red points that were distinctly visible even under low power. Species identification based on morphology also requires analysis of the adult-stage filaria. Unfortunately, our study did not find adult-stage filaria in lions. Nevertheless, the prevalence of microfilaria in lions should be noted and sampled for further analysis.

Routinely, diagnosis is carried out through microscopic examination of the morphology in microfilariae isolated from blood. It is known that *B. malayi* and *B. pahangi* are very similar morphologically. However, species identification by Giemsa staining is not sufficient (43). Although acid phosphatase staining is effective it is not reproducible and the procedure is complicated (44). Molecular methods based on species-specific PCRs are simple and easy to perform and have been introduced for discrimination of *Brugia* sp. (6, 44). Molecular diagnostic methods, based on the amplification of parasite DNA by PCR methods have the advantage of being more sensitive in detecting parasites than the usual microscopy methods, especially in case of low mf densities, as well as increased certainty in the identification of the species or even strain level. This study applied molecular phylogenetic analysis of 12S rDNA, 18S rDNA, COI, and *wsp* nucleotide sequences for confirmation of *Brugia* sp. in a single lion reservoir. In the tree analysis, the phylogenetic tree based on 12S rDNA, 18S rDNA and COI gene sequences were grouped with *B. malayi* sequences from other animals and other countries such as Thailand, Vietnam and USA. Notably, a *B. malayi* isolate obtained from a lion in a Thai zoo exhibited close genetic proximity to isolates from humans and dogs, suggesting a potential reservoir host. While suspicions of *B. malayi* presence in dogs have persisted concerning lymphatic filariasis, reports of *B. malayi* infection in lions were previously nonexistent. Our findings provide evidence for the potential role of lions as reservoir hosts for *B. malayi.* Moreover, there have been reports of *B. malayi* infection in humans in southern Thailand, particularly in regions near the Thai-Malaysian border. Studies have identified *Mansonia* sp. as a significant carrier of infective *Brugia* larvae in these areas, suggesting its role as one of the primary vectors for *B. malayi* in southern Thailand (45, 46). However, conclusive determination of the lion’s reservoir capacity in endemic areas necessitates further on-site investigations involving animals in the vicinity of the zoo.

The primer set COXI-int-F and COXI-int-R employed in our study functioned as universal primers, capable of amplifying the COI region from 11 diverse species of blood and tissue filariae, including *B. malayi*, *B. pahangi*, *D. immitis*, *D. repens*, *Wuchereria bancrofti*, and *Onchocerca* spp. (25). In a 2019 study by Satjawongvanit et al., the detection of filarial nematode DNA in blood samples from domestic dogs in the Bangkok Metropolitan Region, Thailand, was accomplished using COI and internal transcribed spacer 1 (ITS1) gene-based PCR. Based on partial nucleotide sequences of the COI gene (~690 bp), they identified three species in domestic dogs: *D. immitis* (57.89%), *B. pahangi* (22.81%), and *B. malayi* (7.02%). The study emphasized the utility of the COI gene as a valuable marker for distinguishing between *D. immitis*, *B. pahangi*, and *B. malayi*, making COI-based PCR a suitable tool for detecting filarial nematode infections in dogs (47). Additionally, mitochondrial 12S rDNA primers were employed to construct the phylogeny. Although both the 12S rDNA and COI gene were deemed useful markers for detecting filarial nematodes in our study, Gaillard et al. (48) reported that the phylogenetic analyses and amplification of the 12S rDNA gene demonstrated less discriminating power compared to the COI fragment.

The phylogenetic trees of *wsp* gene for *Wolbachia* endosymbiont were distinctly separated into 2 groups *B. malayi* and *B. pahangi*. However, the results of *wsp* showed little differences in nucleotide sequences between the two *Brugia* species with is differs from previous reports by Bazzocchi et al. (49) who showed that the sequences of *B. malayi* and *B. pahangi* were grouped in *wsp* phylogeny. However, all the phylogenetic relationships which are unquestioned for the host nematodes are matched by the *Wolbachia* phylogeny based on *wsp* this indicated that the *Wolbachia*-filaria association is stable and species-specific.

## 5 Conclusion

To the authors’ knowledge, this case represents the initial occurrence of *Brugia* infection in a captive lion from Thailand. The nematode was identified through both morphological and molecular methods, revealing a *B. malayi* infection, which has been documented in domestic animals within Thailand. Nonetheless, our findings bring attention to the insufficient understanding concerning the diversity of *Brugia* species and the interactions between hosts and parasites in wildlife animals from Thailand. This knowledge gap has potential implications in the fields of veterinary medicine and public health.

## Data availability statement

The datasets presented in this study can be found in online repositories. The names of the repository/repositories and accession number(s) can be found in the article/Supplementary material.

## Ethics statement

The requirement of ethical approval was waived by Biosafety Committee of Chulalongkorn University, Faculty of Veterinary Science (IBC 2231033) for the studies involving animals because this study was a part of the routine health check at a private farm, for which ethical approval was not obligatory. All procedures conducted during the study adhered to the relevant guidelines and regulations. The studies were conducted in accordance with the local legislation and institutional requirements. Written informed consent was not obtained from the owners for the participation of their animals in this study because this was a part of the routine health check at Nakhon Ratchasima Zoo, Zoological Park Organization of Thailand, for which ethical approval was not obligatory.

## Author contributions

WJ: Conceptualization, Investigation, Methodology, Validation, Writing – original draft. PK: Investigation, Methodology, Visualization, Writing – original draft. AP: Investigation, Resources, Visualization, Writing – original draft. PT: Writing – original draft, Writing – review & editing.

## Supplementary material

SUPPLEMENTARY FIGURE 1. Phylogenetic tree based on an alignment of COI gene sequences. Bootstrap confidence values (1,000 replicates) are shown as percentages. Values less than 50% are omitted. The units for the scale bar are substitutions per site. NCBI accession numbers are included. The COI sequence of B. malayi from lion in Thailand, with taxon name shown in blue color and bold font, was generated as part of this study. Letters to the right of the bracketed branches denote the clusters. Setaria tundra and S. labiatopapilosa were used as out group.

SUPPLEMENTARY FIGURE 2. Phylogenetic tree based on an alignment of wsp sequences. Bootstrap confidence values (1,000 replicates) are shown as percentages. Values less than 50% are omitted. The units for the scale bar are substitutions per site. NCBI accession numbers are included. The wsp sequence of B. malayi from lions in Thailand, with taxon name shown in blue color and bold font, was generated as part of this study. Letters to the right of the bracketed branches denote the clusters. Wolbachia endosymbiont of Aedes albopictus was used as out group.
