# Supplementary figures and images for: Unveiling zoonotic threats: molecular identification of Brugia sp. infection in a lion
Source: Front Vet Sci. 2024 Apr 12;11:1376208. doi: 10.3389/fvets.2024.1376208 (PMC11047776; doi:10.3389/fvets.2024.1376208)

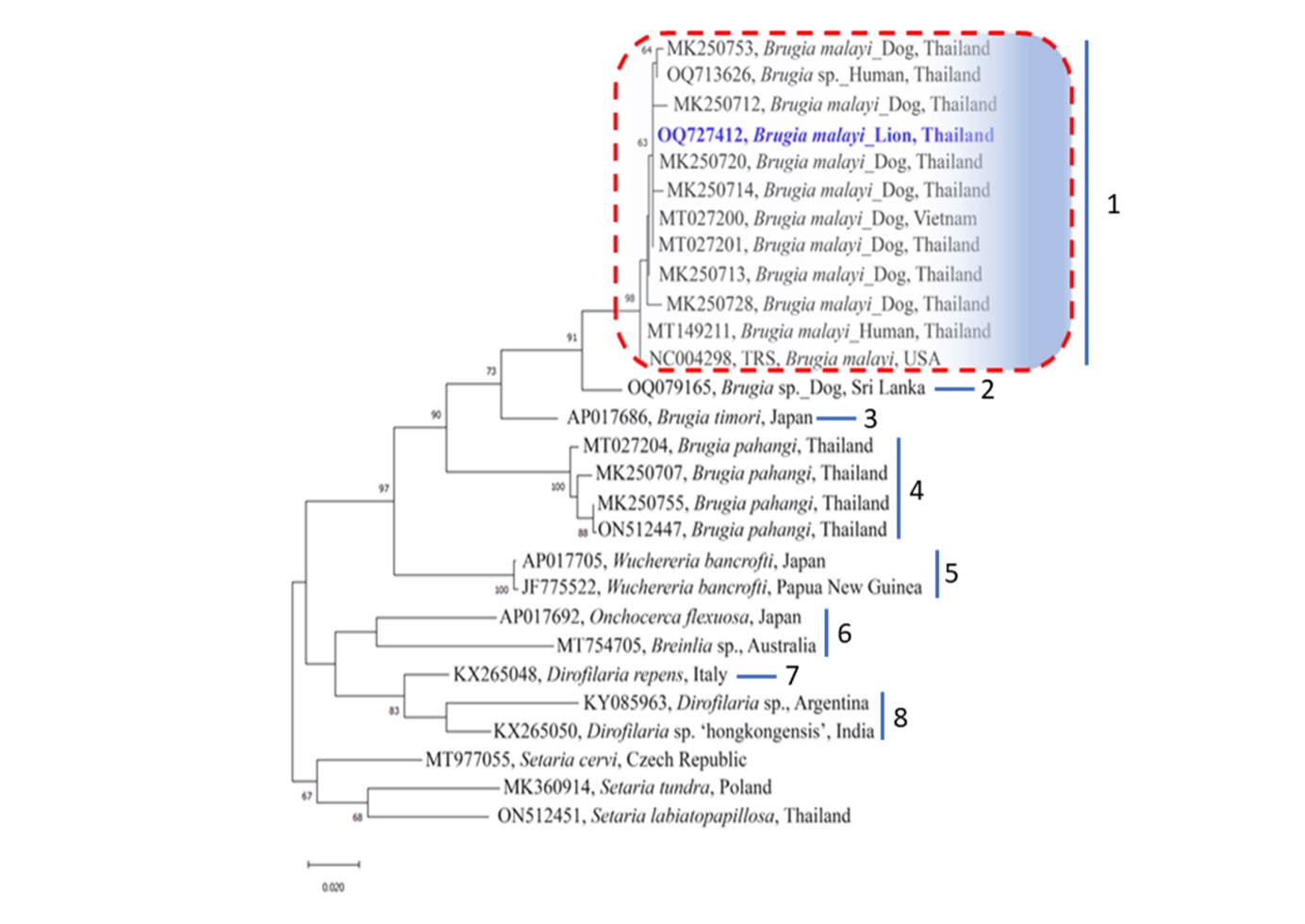

Supplement: SUPPLEMENTARY FIGURE 1. — Phylogenetic tree based on an alignment of COI gene sequences. Bootstrap confidence values (1,000 replicates) are shown as percentages. Values less than 50% are omitted. The units for the scale bar are substitutions per site. NCBI accession numbers are included. The COI sequence of B. malayi from lion in Thailand, with taxon name shown in blue color and bold font, was generated as part of this study. Letters to the right of the bracketed branches denote the clusters. Setaria tundra and S. labiatopapilosa were used as out group. [file Image_1.jpg]

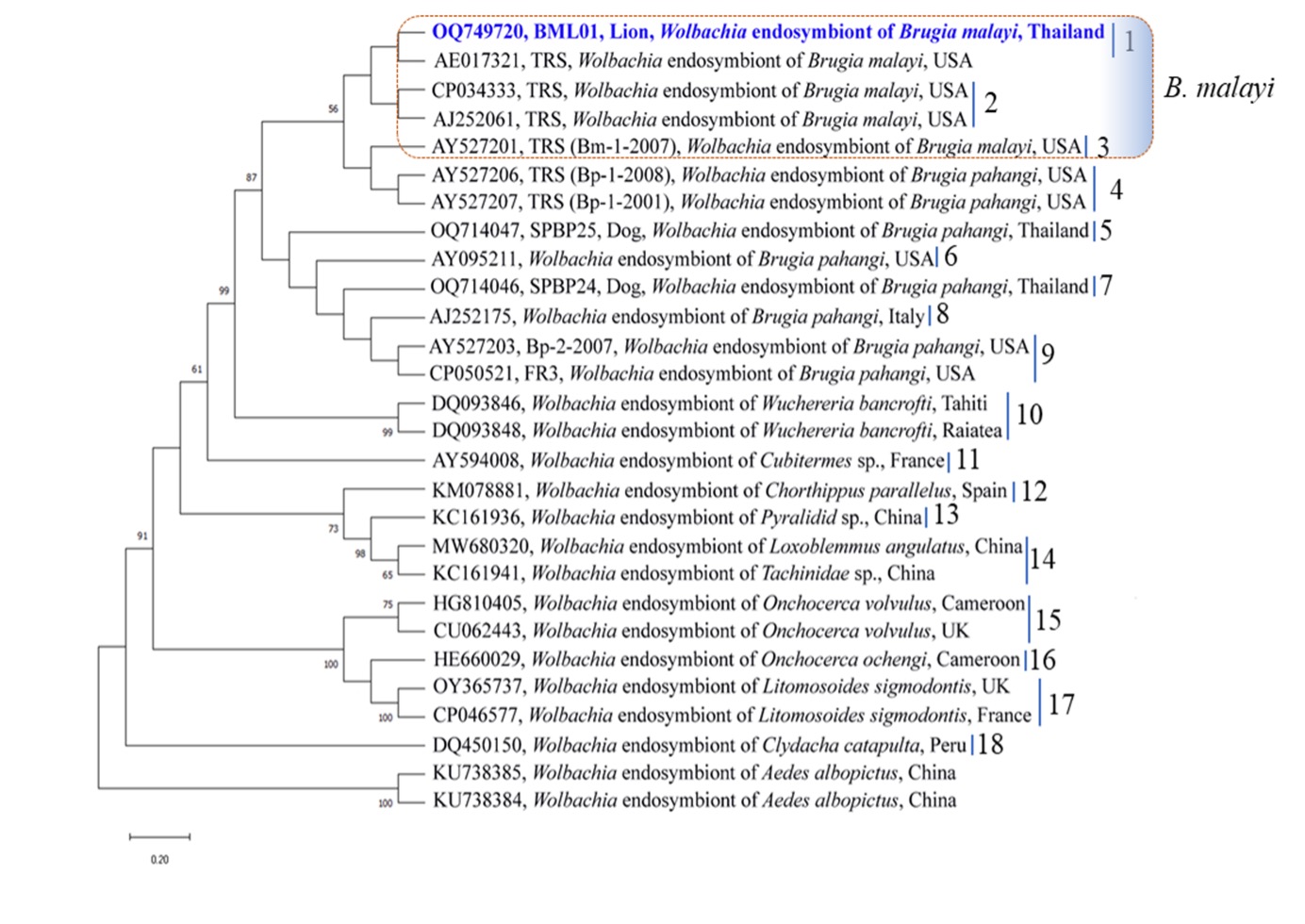

Supplement: SUPPLEMENTARY FIGURE 2. — Phylogenetic tree based on an alignment of wsp sequences. Bootstrap confidence values (1,000 replicates) are shown as percentages. Values less than 50% are omitted. The units for the scale bar are substitutions per site. NCBI accession numbers are included. The wsp sequence of B. malayi from lions in Thailand, with taxon name shown in blue color and bold font, was generated as part of this study. Letters to the right of the bracketed branches denote the clusters. Wolbachia endosymbiont of Aedes albopictus was used as out group. [file Image_2.jpg]
